# Existential Vacuum and External Locus of Control as Predictors of Burnout among Nurses

**DOI:** 10.3390/nursrep11030053

**Published:** 2021-07-16

**Authors:** Othman A. Alfuqaha, Yazan Al-olaimat, Ahmad Sami Abdelfattah, Rand Jamal Jarrar, Bashar Mazin Almudallal, Zaid Ibrahim Abu ajamieh

**Affiliations:** 1 Department of Nursing, Jordan University Hospital, The University of Jordan, Amman 11942, Jordan; 2 School of Medicine, Jordan University Hospital, The University of Jordan, Amman 11942, Jordan; Olimat_1989@yahoo.com (Y.A.-o.); Ahmed_sami_r@yahoo.com (A.S.A.); Rand.jarrar@yahoo.com (R.J.J.); bashar.mudallal@gmail.com (B.M.A.); Zaidabuajamieh1@yahoo.com (Z.I.A.a.)

**Keywords:** existential vacuum, locus of control, nurses, psychological burnout

## Abstract

Existential vacuum and psychological burnout are becoming increasingly important issues in healthcare professions, especially nursing. This study aimed to investigate the contribution of several demographic factors including gender, work position, experience, and educational level as well as existential vacuum and locus of control (external and internal) in predicting burnout among nurses. A convenience sample of 181 nurses was selected to represent the study sample. Participants were assessed using an existence scale, locus of control scale, and burnout scale. The study showed that 40.3% of nurses had severe existential vacuum. It was found that 93.9% of nurses had experienced a moderate level of burnout. External locus of control was the most common personality trait among participating nurses in this study. It also was found that existential vacuum and external locus of control were the main predictors of psychological burnout among nurses. The findings of our study highlight major problems facing nursing, such as existential vacuum and psychological burnout. It is recommended to enhance nurses’ workplace, provide proper psychological prevention programs, and teach advocacy skills.

## 1. Introduction

Existential fulfillment and psychological burnout are becoming increasingly important issues among healthcare professions, especially nursing. Nursing as a humanistic job may be subject to many psychological problems due to significant job demands, lack of job resources, and work disengagement [1]. Hence, researchers in nursing and psychology need to assess many psychological factors in workplaces, such as work environment and personal resources to enhance nurses’ occupational health and overall wellbeing [2].

Frankl, who developed logotherapy as a form of psychotherapy, guided people to identify their purposes in life and take responsibility for their own choices to form a good life. Existential analysis deals with the meaning of one’s life, which is considered one of the basic human needs [3]. Nowadays, existential fulfillment (EF) is considered one of the most important concepts in existential psychology, and refers to life led with meaning, full of goals, and with the feeling of mattering [4]. Existential vacuum is the opposite of EF and can be defined as loss of meaning and interest in life. Langle, the founder of the Viennese school of existential analysis, developed with his co-workers (2003) four major processes in EF called Personal Existential Analysis (PEA). These processes, which include self-distance, self-transcendence, freedom, and responsibility, are all responsible for creating personalities and the realities of life [3,5].

Due to the nature of the workplace as a part of life, nurses search for meaning in their jobs (self-distance), and if they fail to make a clear differentiation between themselves and the workplace, they may suffer from psychological distress such as anxiety, loneliness, depression, anger, irritability. This can ultimately lead to existential vacuum. Self-transcendence is the second process of PEA, which is the core element in EF; in self-transcendence, one considers the relationship between reality and his/herself, and if the relationship is based on reality and logic, they will reach to the level of experience [6]. Freedom is an ability to do what one wants without constraint and to choose one’s attitude, which can lead individuals to be more aware of their decisions and their consequences. Responsibility for one’s actions and decisions is the fourth process of PEA [3].

Locus of control is a personality trait detecting the degree to which people believe that they have control or no control over the outcome of their situations in life. Rotter divided the locus of control into two major parts: external and internal. External locus of control explains that control is related to outer forces such as destiny, luck, and people, which indicates the individual is not responsible for his/her actions. Conversely, internal locus of control explains that control is related to one’s own ability, behavior, and effort, which makes the individual responsible for his/her actions [7]. The effects of internal and external locus of control among nurses have been reported to have positive and negative outcomes. Internal locus of control has been determined to have positive outcomes such as nurses having less job stress [8,9], being more ready to self-direct learning [10], and experiencing more empowerment [11]. On the other hand, external locus of control has been demonstrated to have negative outcomes among nurses, such as a higher level of burnout in Spain [12], procrastination in Iran [13], and a lower satisfaction level with their jobs [14]. In the literature, little attention has been paid to locus of control among nurses.

Psychological burnout is a state of prolonged job stressors in workplace recognized when individual begins to dislike their work; the relationship fails, leading to lack of energy, feelings of exhaustion, and loss of meaning at work. Psychological burnout has been recently defined by the World Health Organization as prolonged stressful events in the workplace that have been inappropriately managed [15]. However, feelings of inner fulfillment in the workplace play an important role in preventing nurses from experiencing psychological distress, particularly physical and emotional exhaustion, which are the main elements in burnout [16,17]. Burnout is also affected by negative attitudes towards life and feelings of hopelessness and meaninglessness in the workplace [18,19]. Due to burnout, nurses have been affected physically and mentally. It has been reported that burnout is negatively correlated with EF [20]. Tomic and Tomic [21] indicated that EF was an important burnout determinant among principals and teachers. On the other hand, Tomic [22] found that the higher the levels of workload among city council centers, the lower the scores in EF and work engagement. Finally, Alfuqaha et al. [23] found that self-evaluation and professional status play an important role as predictors of burnout among nurses in Jordan. The existential vacuum of nurses has been not investigated well.

Existential vacuum leads to many psychological and emotional instabilities, isolation, anxiety, depression, and dysfunctions that stem from the workplace [6,20]. However, to the best of our knowledge, there is only a limited body of work in the current research that focuses on existential vacuum and locus of control among nurses. Therefore, in an attempt to study life meaning and the purpose of life among nurses’ workplaces, and their impact on psychological burnout, this study aimed to investigate the contribution of several demographic factors including gender, work position, experience, and educational level as well as existential vacuum and locus of control (external and internal) in predicting burnout among nurses. Furthermore, it aimed to determine the level of existential vacuum, locus of control (external and internal), and burnout among nurses in Jordan.

## 2. Materials and Methods

### 2.1. Study Design

The study used a cross-sectional design to achieve its aims.

### 2.2. Participants and Settings

Participants were nurses in Jordan University Hospital (JUH) at Amman-Jordan during the period of January to February 2019. This hospital was chosen since it is readily accessible for the researchers and is the biggest hospital in Amman (600 beds), which requires sufficient nursing staff of the selected hospital. A total sample size of 100–300 nurses was determined to be necessary to achieve a margin of error of 5% and confidence level of 95% [24]. In this regard, a convenience sample of 300 nurses was selected to participate in this study. Inclusion criteria were nurses at all units and departments in the selected hospital who had a sufficient educational level, experience in nursing of at least 1 year, engaged in regular work (not part-time) and agreed to participate. Exclusion criteria were nurses working as supervisors and managers. The questionnaire consisted of demographic data (i.e., gender, work position, and educational level) and three scales to measure existential vacuum, locus of control, and psychological burnout. We distributed 300 questionnaires to nurses who met all inclusion criteria, taking into consideration the nurses’ different shifts (day/night). A total of 181 participants completed the questionnaire for a response rate of 60.3%. The other 119 participants either refused, were not interested, or were found to have turned in incomplete questionnaires. We explained the aims of the study to all participants. Ethical guidelines were followed, and nurses were free to choose whether to participate or not. Before collection of the data, ethical approval was sought, and subsequently provided, from the Institutional Review Board in the selected hospital (No. 10/2019/28025).

### 2.3. Study Scales

All selected scales were translated to the Arabic language, and validity and reliability were checked before being used. The three scales were used to represent existence scale, locus of control, and psychological burnout as described below.

#### 2.3.1. Existence Scale

The Existence Scale (ES), developed by Langle and his colleagues [3], consists of 46 items (8 items pertaining to self-distance, 14 items related to self-transcendence, 11 items regarding freedom, and 13 items concerning responsibility). Psychometric properties for the Arabic version of the ES were assessed among nurses in Jordan. The Arabic translation of the ES utilized for this study was found to be valid and reliable in 37 items (4 constructs); this is currently under consideration for publication elsewhere. The four constructs can be clarified as follows: first, the 8- item self-distance construct assesses the events and situations that happen in the workplace; second, the self-transcendence construct describes the relationship between reality and the self to reach a satisfactory conclusion in the workplace and contains 9 items; third, the freedom construct assesses the ability to act freely without constraints and contains 10 items; and fourth, the responsibility construct refers to awareness of decisions and contains 10 items. To measure the sense of existential vacuum and loss of meaning in life among nurses, we classified as existential vacuum as severe (1 to 1.99), moderate (2–2.99), or mild (3 to 4). ES was rated on a 4-point Likert-type scale as follows: 1 ‘Strongly Agree’, 2 ‘Agree’, 3 ‘Disagree’, and 4 ‘Strongly Disagree’ for positive items; negative items were evaluated in a reversed order. A high score of the ES is related to high existential fulfillment, while a low score is related to existential vacuum and evidences a sense of loss of meaning and purpose in life. Cronbach’s alpha for the ES was 0.93, and for the four constructs, ranged from 0.88 to 0.93.

#### 2.3.2. Locus of Control Scale

The Locus of Control Scale (LCS) consists of 22 items which depend on the Rotter scale [7]. The LCS is divided into two major sections as follows: 11 items for the assessment of external locus of control refers to nurses that believe control is related to outside forces such as destiny, luck, and other people. The second dimension is internal locus of control, which consists of 11 items and asks question about how nurses have been feeling related to their own ability, behavior, and effort. Each of the 22 items were rated as follows: Yes = 1, No = 0, for the items pertaining to external locus of control, while the items pertaining to internal locus of control were rated on a reversed order where Yes = 0, No = 1. The LCS was applied to 30 nurses out of the study sample and the correlation coefficient between the items of LCS ranged between 0.55–0.90. Cronbach’s alpha for the external LCS was 0.63, and Cronbach’s alpha was 0.68 for the internal LCS.

#### 2.3.3. Burnout Scale

The Maslach Burnout Inventory (MBI) was adopted in this study. The original MBI consisted of 22 items divided into 3 subscales; 9 items in the emotional exhaustion subscale pertain to feelings of being physically and emotionally exhausted; 5 items in the depersonalization subscale describe negative feelings; and 8 items in the personal accomplishment subscale measure low personal achievements [25]. There was an optional subscale (3-items) on the original MBI; these items were added for a total of 25 items. Accordingly, we tested the psychometric properties of these 25 items among nurses in Jordan. Translation process, face, content, construct, convergent, and discriminant validity were checked. Moreover, Cronbach’s alpha and test-retest were used to assess the reliability of the MBI.

To begin with, the translation process was checked by four independent experts in counseling, psychology, nursing, and psychiatry throughout two steps: translation and backtranslation. In conclusion, experts were on the final version of the Modified MBI. To check face validity, a pilot study of 30 nurses was completed and, therefore, none of the items were removed. Content validity was assessed through 10 arbitrators in the related field. Acceptance of at least 80% of arbitrators on the Modified MBI items was mandatory to achieve content validity index [26]. All items scored greater than the acceptance rate of 80%. To determine the construct validity, explanatory factor analysis was checked by principal component analysis (varimax rotation). Values greater than 0.40 of factor loading was assumed, eigenvalues greater than 1 were considered construct. Kaiser-Meyer-Olken (KMO) greater than 0.70 was considered superior, and finally, Bartlett’s test of sphericity was considered to be significant [27]. Three factors were loaded with eigenvalues greater than 1. A total of 9 items was loaded in the emotional exhaustion subscale, ranging from 0.63 to 0.89 of factor loadings; 7 items were loaded in the depersonalization subscale from 0.58 to 0.87; and 9 items were loaded in the personal accomplishment subscale ranging between 0.62 and 0.84. The three constructs of the Modified MBI collectively explained 61.65% of the variance. The KMO test was found to be 0.80. Bartlett’s test of sphericity was significant (*p* < 0.01). Thus, explanatory factor analysis showed that the Modified MBI is valid among nurses in Jordan. Finally, to assess discriminant and convergent validity, inter-correlation between the exogenous constructs of the Modified MBI (emotional exhaustion, depersonalization, and personal accomplishment), composite reliability, average variance extracted, and the square root of the average variance extracted were calculated. Results are presented in Table 1.

Table 1 reveals that the discriminant validity of the Modified MBI achieved the criteria, since composite reliability is above 0.70 for all constructs [28]. Moreover, the square root of the average variance extracted was higher than the inter-construct correlations, indicating convergent validity of the Modified MBI [27]. Regarding reliability, Cronbach’s alpha and test-retest were both above 0.60, therefore indicating that the Modified MBI is reliable among nurses in Jordan [29].

The Modified MBI was rated on a 5-point Likert-type scale as follows: ‘Never = 1’, ‘Rarely = 2’, ‘Sometimes = 3’, ‘Frequently = 4’, and ‘Always = 5’. High scores of the mean were considered severe burnout (3.68 to 5), moderate mean scores were considered moderate burnout (2.34 to 3.67), and low mean scores were considered mild burnout (1 to 2.33).

### 2.4. Data Analysis

The Statistical Package for the Social Sciences (SPSS v. 22) was utilized for analyzing the results. The normality test, in the form of the Kolmogorov–Smirnov test, was checked before using the data. To the first aim of this study, descriptive statistics, t-test, and F-distribution were used. Linear regression (stepwise) was utilized to investigate several demographic factors as well as existential vacuum and locus of control in predicting burnout among nurses in Jordan.

## 3. Results

### 3.1. Association of Demographic Factors on Burnout

A total of 181 participants was recruited in this study with a response rate of 60.3%. To examine the association of sociodemographic variables on the occurrence of burnout among nurses in Jordan, frequencies, means, standard deviations, t-test, one-way ANOVA, and significant levels were calculated and presented in Table 2.

Table 2 shows that two-thirds of the participants were female and had a bachelor’s degree. A total of 37.6% of participating nurses were working for the surgical ward, and 26.5% were working for intensive care units. Generally, moderate levels of burnout were shown among nurses’ socio-demographic factors. None of the demographic factors were significantly associated with the occurrence of burnout among participated nurses in this study.

### 3.2. Levels of the Selected Variable

To illustrate the levels of the selected variable in this study, means, standard deviations, and overall levels of all variables are presented in Table 3.

We went in-depth to assess the selected variables according to our standard cut-off scores; results are described in Table 4.

Table 3 shows that the total mean score of existential vacuum was (2.10 ± 0.35) among participating nurses, thereby reflecting a moderate level of existential vacuum. The self-distance construct exhibited the lowest mean score in ES, indicating severe existential vacuum among participated nurses. Collectively, all constructs of ES were shown to be at the moderate level. Table 3 demonstrates a mean score of (0.53 ± 0.18) for internal locus of control; this score was (0.60 ± 0.17) for external locus of control. However, the *t*-test exhibited a statistically significant difference between the internal and external locus of control among participating nurses in Jordan (M_external_ = 0.60, M_internal_ = 0.53, t = 3.80, *p* < 0.001). Additionally, Table 3 reveals the prevalence of the total level of psychological burnout and has a mean score of (3.21 ± 0.36), thereby indicating a moderate level of burnout. Emotional exhaustion displays the highest mean score, indicating that a severe level of emotional exhaustion. Other constructs of burnout have been demonstrated to be at the moderate level.

The prevalence of severe existential vacuum and burnout in participating nurses was 40.3% and 5.5% respectively. Almost ninety-four (94%) of participating nurses stated they had moderate levels of psychological burnout.

### 3.3. Predictors of Psychological Burnout

To investigate the contribution of several demographic factors including gender, work position, experience, and educational level as well as existential vacuum and locus of control (external and internal) in predicting burnout, step wise regression was run, and the results are presented in Table 5.

Two models were built in Table 5. These models suggested that existential vacuum and external locus of control were the main predictors of burnout among participating nurses in this study, with a total variation of 68.2%. Existential vacuum alone explained 58.4% of the variance, while the external locus of control explained 9.9% of burnout. All demographic factors, as well as internal locus of control, were excluded in this model.

## 4. Discussion

This study aimed to investigate the contribution of several demographic factors including gender, work position, experience, and educational level as well as existential vacuum and locus of control (external and internal) in predicting psychological burnout among nurses. Furthermore, it aimed to determine the level of existential vacuum, locus of control (external and internal), and psychological burnout among nurses in Jordan.

The decrease level in self-distance and the increase level in emotional exhaustion depended on several factors such as the workplace of nurses, time pressure, the surrounding environment, and imbalanced needs, and may reflect on how nurses explained the events and situations that happened in the workplace.

Nurses have a serious existential vacuum that can negatively affect their personal life as well as their work life. It was observed that most participants suffered from existential vacuum which leaded to severe levels of psychological burnout. The higher the existential vacuum level of a nurse the more prone he/she is to psychological burnout. These results can be explained by the participating nurses’ experiences with feelings of hopelessness, unhappiness in doing their job, unsupportive environment, lack of inspiration and motivation, loss of interest, and emotional fatigue, or may be related to conflicts between colleagues, supervisors, and administrators as a whole. Previous studies have revealed that burnout among nurses has significant effects on their satisfaction level, self-efficacy, and depression [30,31].

We found that nurses have more significant external locus of control than internal locus of control. This type of personality, which depends on outer forces, destiny, and luck, is the primary source of psychological burnout among nurses. It was observed that nurses in our study believed in outer forces such as managers and directors to handle situations; hence, they have poor autonomy and control over their workplace as well as their decisions. This result is in agreement with the study of [13], but disagrees with the study of [10], where researchers found that the internal locus of control was the most common personality type among nurses in Turkey.

Severe emotional and physical exhaustion was observed among participating nurses. This is a result of an excessive workload, a shortage of nurses, suffering from time pressure, and poor relationships with colleagues. In addition, emotional exhaustion has negative effects on the meaning and purpose of life. The high level of psychological burnout among nurses is well-documented in previous studies [32,33].

Existential vacuum and external locus of control were found to be the primary sources of psychological burnout among nurses. Nurses are involved with several critical cases that cause loss of control, feelings of hopelessness, and meaninglessness. Lack of social support, low professional status, agreeableness, depression, and low self-evaluation are the main predictors of burnout among nurses [23,34,35]. Similar to our findings, previous studies have shown that existential fulfillment was negatively associated with psychological burnout [19,36]. Existential vacuum scores among nurses were higher in nurses with severe psychological burnout. Finally, other demographic factors which were included in this study were not found to be associated with burnout among nurses. One of the solutions to solve the problem of burnout among nurses is the job rotation approach. This approach helps nurses to have job satisfaction and commitment in their workplace [37].

Some limitations in our study were found such as the fact that one hospital was selected to participate in this study. Other limitations included total sample size (181 nurses) and scales that were used in this study, but this study highlights major problems facing nursing such as existential vacuum and psychological burnout.

## 5. Conclusions

The study showed that 40.3% of nurses had severe existential vacuum. It was found that 93.9% of nurses had experienced a moderate level of burnout. Emotional exhaustion and self-distance were found to be the main psychological problems in nurses. External locus of control was the most common personality trait among participating nurses in this study. It also was found that existential vacuum and external locus of control were the main predictors of psychological burnout among nurses. We recommend enhancing the nurses’ workplace, providing proper psychological prevention programs, applying the job rotation approach, and teaching advocacy skills in order to improve their life meaning and their overall wellbeing. Further studies in existential vacuum are recommended in different healthcare professions such as physicians and physiotherapists.

## Figures and Tables

**Table 1:** Inter-correlation between constructs, composite reliability, average variance extracted, and test-retest results for the Modified MBI.

Construct	EE	DP	PA	CR	AVE	Alpha	Test-Retest
Emotional exhaustion (EE)	**0.77**			0.93	0.59	0.82	0.80
Depersonalization (DP)	0.55	**0.67**		0.91	0.45	0.86	0.84
Personal accomplishment (PA)	0.49	0.39	**0.75**	0.92	0.56	0.89	0.81

CR: Composite reliability. AVE: Average variance Extracted. Alpha: Cronbach alpha. Note: Diagonal in the bold type presents the square root of the average variance extracted.

**Table 2:** Prosperities and the occurrence of burnout among nurses’ socio-demographic factors (*n* = 181).

Demographic Data	Descriptive	Frequency (%)	M ± SD	*t*-Test/F-Distribution	*p*-Value
Gender	Male	61 (33.7)	3.22 ± 0.28	0.46	0.64
	Female	120 (66.3)	3.20 ± 0.40		
Work position	Surgical Ward	68 (37.6)	3.20 ± 0.34		
	Medical Ward	43 (23.8)	3.15 ± 0.37	0.65	0.58
	Intensive Care Unit	48 (26.5)	3.26 ± 0.43		
	Emergency Room	22 (12.2)	3.21 ± 0.28		
Experience (Years)	1–5 Y	85 (47)	3.22 ± 0.46	0.36	0.7
	6–10 Y	57 (31.5)	3.17 ± 0.26		
	≥11 Y	39 (21.5)	3.23 ± 0.30		
Educational level	Diploma Degree	33 (18.2)	3.19 ± 0.32		
	Bachelor’s Degree	120 (66.3)	3.21 ± 0.40	0.03	0.96
	Postgraduate Degree	28 (15.5)	3.21 ± 0.36		

M ± SD: Mean ± standard deviation.

**Table 3:** Descriptive statistics for the dimensions of existential vacuum, dimensions of locus of control, and dimensions of burnout among nurses (*n* = 181).

Variables	Construct	M ± SD	Overall Level
Existential vacuum	Self-distance	1.85 ± 0.42	Severe
	Self-transcendence	2.15 ± 0.47	Moderate
	Freedom	2.19 ± 0.47	Moderate
	Responsibility	2.23 ± 0.40	Moderate
	Total	2.10 ± 0.35	Moderate
Locus of control	Internal locus of control	0.53 ± 0.18	-----
	External locus of control	0.60 ± 0.17	-----
Psychological burnout	Emotional exhaustion	3.74 ±0.49	Severe
	Depersonalization	3.35 ± 0.93	Moderate
	Personal accomplishment	2.56 ± 0.60	Moderate
	Total	3.21 ± 0.36	Moderate

M ± SD: Mean ± standard deviation.

**Table 4:** Prevalence of existential vacuum and psychological burnout among nurses (*n* = 181).

Variables	Descriptive	*n*	*%*
Existential vacuum	Severe existential vacuum (1–1.99)	73	40.3
	Moderate existential vacuum (2–2.99)	105	57.9
	Mild existential vacuum (3–4)	3	1.8
Psychological burnout	Severe burnout (3.68–5)	12	5.5
	Moderate burnout (2.34–3.67)	168	93.9
	Mild burnout (1–2.33)	1	0.6

**Table 5:** Results of regression analysis (stepwise regression) for burnout predictors.

Model	R	R²	R Change	Unstandardized Coefficients		*t-*Test	*p*-Value
				B	Std. Error		
1 ^a^	0.764	0.584	0.584	2.209	0.065	33.813	<0.001 **
				0.297	0.019	−15.837	<0.001 **
				1.437	0.119	12.125	<0.001 **
2 ^b^	0.826	0.682	0.099	−0.255	0.017	−14.638	<0.001 **
				−0.245	0.033	−7.438	<0.001 **

^a^ Predictors: (Constant), Existential vacuum. ^b^ Predictors: (Constant), Existential vacuum, External locus of control. ** Significant at *p* ≤ 0.01 level.

## Data Availability

Restrictions apply to the availability of these data. Data was obtained from Jordan University Hospital and are available from the corresponding authors with the permission of Jordan University Hospital.
